# Carbohydrate Intake Levels and the Risk of Metabolic Syndrome in Korean Populations: A Prospective Study

**DOI:** 10.3390/nu16152440

**Published:** 2024-07-26

**Authors:** Hyeonji Yoo, Unhui Jo, Kyong Park

**Affiliations:** Department of Food and Nutrition, Yeungnam University, Gyeongsan 38541, Republic of Korea; hyeonjiyoo@yu.ac.kr (H.Y.); unhui_jo@yu.ac.kr (U.J.)

**Keywords:** carbohydrate, metabolic syndrome, Korea, cohort study

## Abstract

In Korea and other Asian countries, traditional high-carbohydrate diets are increasingly associated with metabolic syndrome (MetS) and its complications. As dietary patterns shift, there is a growing need to assess the effect of these changes on health outcomes related to MetS. This study aimed to investigate the prospective relationship between carbohydrate consumption and the risk of MetS and its components. We analyzed data from 7902 participants from the Korean Association Resource, part of the Korean Genome and Epidemiology Study Dietary intakes, including carbohydrates and fiber, were assessed using a validated semi-quantitative food frequency questionnaire, allowing for the calculation of the proportion of total energy from carbohydrates (P_CARB) and the carbohydrate-to-fiber ratio to assess carbohydrate quality. Blood samples were collected after at least eight hours of fasting for laboratory analysis. We employed Cox proportional hazards models to estimate hazard ratios and 95% confidence intervals, focusing on the relationship between the P_CARB and the risk of developing MetS and its individual components, while adjusting for carbohydrate quality. In the fully adjusted model, which accounted for carbohydrate quality as a covariate, individuals in the highest percentile of the P_CARB showed a significantly increased risk of MetS, hypertriglyceridemia, hypo-high density lipoprotein cholesterolemia, dyslipidemia, and high blood pressure, compared to those in the lowest P_CARB group. Spline curve analyses indicated that the risks for MetS and its components consistently escalated with increasing P_CARB, with all *p*-values for nonlinearity exceeding 0.05. The findings suggest that higher levels of P_CARB are associated with an increased risk of MetS and related conditions, except for high fasting glucose. These results highlight the importance of dietary awareness and potential adjustments for populations consuming high-carbohydrate diets.

## 1. Introduction

Metabolic syndrome (MetS), characterized by central obesity, insulin resistance, hypertension, and hyperlipidemia, is a significant epidemiological concern in Korea [1]. The “Metabolic Syndrome Fact Sheet in Korea 2021” reports an increase in MetS prevalence from 21.6% in 2007 to 22.9% in 2018 [1], highlighting the urgent need to investigate modifiable risk factors, particularly dietary patterns endemic to Korea.

Carbohydrates, especially white rice and noodles, are staple foods in the Korean diet, particularly among older adults [2,3]. Previous studies have suggested that traditional high-carbohydrate diets in Korea and other Asian countries are increasingly associated with MetS and its complications. As dietary patterns shift, there is a growing need to assess the effect of these changes on health outcomes related to MetS. Despite the prevalence of these dietary habits, there is a lack of longitudinal studies on their long-term effects on MetS. Existing research is primarily cross-sectional [4,5,6,7,8,9], providing limited insight into the chronic impact of dietary habits.

Beyond its medical implications, MetS poses a socio-economic challenge, with rising prevalence leading to higher healthcare costs and a greater burden on the healthcare system due to related complications [10]. As Korea’s population ages, understanding and modifying lifestyle factors, especially diet, becomes crucial.

To address the rising prevalence of MetS and the lack of longitudinal studies on the impact of high-carbohydrate diets, our research investigates the association between carbohydrate consumption and the risk of developing MetS in the Korean population. Leveraging data from the Korean Genome and Epidemiology Study (KoGES), we provide a comprehensive analysis of how both the quantity and quality of carbohydrate intake contribute to the risk of MetS and its components over time. This research seeks to fill existing gaps in the literature and enhance our understanding of how dietary patterns influence the development of MetS, addressing the critical need for evidence-based dietary recommendations in Korea.

## 2. Materials and Methods

### 2.1. Data Source and Participants

This study utilized data from the Korean Association Resource (KARE), part of the KoGES, a comprehensive initiative exploring genetic and environmental factors in chronic diseases prevalent in Korea [11]. The KARE cohort consists of 10,030 middle-aged adults (aged 40–69 years) from Ansan (urban, *n* = 5012) and Ansung (rural, *n* = 5018). Baseline surveys were conducted in 2001–2002, with biennial follow-up surveys extending to the eighth follow-up in 2017–2018 [11].

**Figure 1:**
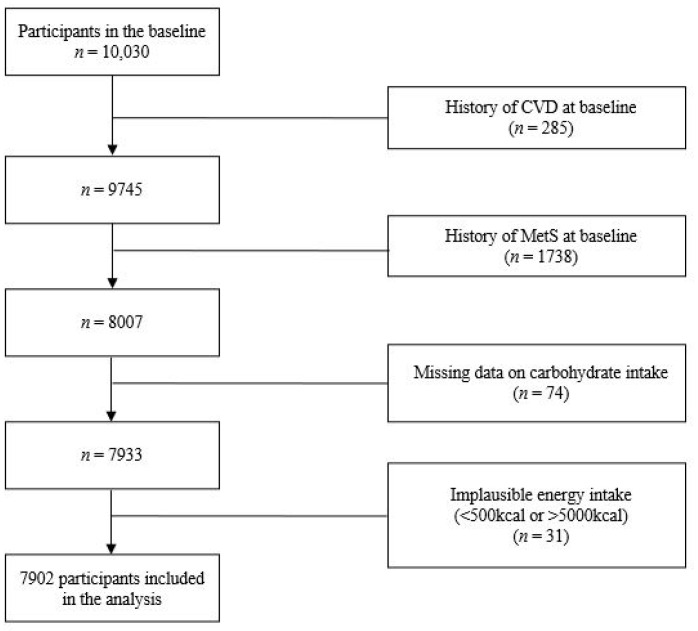
Flow chart of the participants in this study. CVD, cardiovascular disease; MetS, metabolic syndrome.

Participants were excluded if they had: (1) a diagnosis of cardiovascular disease (CVD) such as myocardial infarction, angina, stroke, or were using relevant medications at baseline (*n* = 285); (2) presence of MetS at baseline (*n* = 1738); (3) missing data on carbohydrate intake (*n* = 74); and (4) implausible energy intake, defined as less than 500 kcal or greater than 5000 kcal (*n* = 31). This left 7902 participants for analysis (Figure 1).

The study protocol was approved by the Institutional Review Board (IRB) of the Korea Disease Control and Prevention Agency and Yeungnam University (IRB number: 202112011-UE003, approved on 18 December 2021).

### 2.2. Demographic and Lifestyle Information

In the KARE study, demographic and lifestyle information such as sex, age, household income, smoking status, alcohol consumption, and physical activity were collected through questionnaires [11,12]. Participants were grouped into three age categories: 40–49, 50–59, and ≥60 years. Monthly household income was calculated from the total income of all family members, including any external financial support. Smoking status was classified into smokers (current and occasional) and non-smokers (former and never). Alcohol consumption was categorized into drinkers (current) and non-drinkers (past and never).

Physical activity was quantified using metabolic equivalents-hours per week (METs-h/week) and grouped into three levels based on tertiles of the distribution: low, moderate, and high [13]. Anthropometric measurements included height and weight. Height was recorded to the nearest 0.1 cm with participants standing upright on a flat surface, and body weight was measured to the nearest 10 g with participants wearing light clothing [14]. Body mass index (BMI) was calculated by dividing weight (kg) by height squared (m^2^). 

### 2.3. Dietary Information 

Dietary data were collected using a validated semi-quantitative food frequency questionnaire (SQFFQ), designed to assess the frequency and amount of commonly consumed food items over the past year [11,15,16]. The frequency of consumption was categorized into nine levels, ranging from ‘rarely’ to ‘3 times/day’, with portion sizes classified as small, medium, and large [11]. 

The baseline survey included 103 food and recipe items, based on the 1998 Korea National Health and Nutrition Examination Survey, which was updated to 106 items in 2006. For our analysis, we averaged the dietary data from the baseline and second follow-up surveys. 

The proportion of total energy from carbohydrates (P_CARB) was calculated using the formula: ([carbohydrate intake (g) × 4] ÷ [total energy intake (kcal)]) × 100. To assess the quality of carbohydrate consumption, we also calculated the carbohydrate-to-fiber ratio.

### 2.4. Blood Sample Collection and Analysis

Trained investigators explained the purpose of genetic testing and obtained informed consent from participants in accordance with the Bioethics and Safety Act. Blood samples were collected after participants had fasted for 12 hours [17]. The collection involved using a serum separator tube (SST; 8.5 mL) and two ethylenediaminetetraacetic acid (EDTA) tubes (10 mL and 3 mL). Blood samples were collected with participants in a sitting position. The protocol for blood collection was strictly followed to ensure the safety of participants and the integrity of samples [11]. 

Clinical chemistry tests were conducted using different instruments over the study period due to equipment upgrades: HITACHI 7600 (Hitachi, Chiyoda, Tokyo, Japan) until 31 August 2002, ADVIA 1650 (Siemens, Washington, DC, USA) until 31 January 2011, and ADVIA 1800 (Siemens, Washington, DC, USA) from 21 February 2011 [17]. 

### 2.5. Definition of MetS and Its Components

In our study, MetS was defined using criteria adapted from the Korean Society of Lipidology and Atherosclerosis and the National Cholesterol Education Program Adult Treatment Panel III, with specific adjustments for the Korean context [18,19]:

- Hypertriglyceridemia: Triglyceride levels ≥ 200 mg/dL.
- Hyperglycemia: Fasting glucose ≥ 100 mg/dL or use of diabetes medication/insulin
- High Blood Pressure: Systolic blood pressure ≥ 130 mmHg, diastolic blood pressure ≥ 85 mmHg, or use of antihypertensive medication
- Abdominal Obesity: Waist circumference > 90 cm in men and > 85 cm in women
- Hypo-high density lipoprotein (HDL) Cholesterolemia: HDL cholesterol < 40 mg/dL

Participants meeting three or more of these criteria were diagnosed with MetS. For additional analyses, dyslipidemia was defined as having a low density lipoprotein (LDL) cholesterol level of 130 mg/dL or higher, total cholesterol of 240 mg/dL or higher, hypo-HDL cholesterolemia, hypertriglyceridemia, or the use of lipid-lowering medication. LDL cholesterol was calculated using the Friedewald formula [20]. 

### 2.6. Statistical Analysis

Demographic characteristics and lifestyle factors collected at baseline were compared across quartiles of the P_CARB. Differences in categorical variables were assessed using chi-square tests, with results presented as frequencies and percentages. The follow-up duration for each participant was calculated from the baseline survey date to the onset of disease or the final survey date for those who did not develop a disease or were lost to follow-up.

Cox proportional hazards regression models were used to examine the association between the P_CARB levels and the incidence of MetS and its components, with hazard ratios (HRs) and 95% confidence intervals (CIs) calculated. A literature review and preliminary analysis identified potential confounding factors such as demographic, lifestyle, and dietary variables that could affect the relationship between the P_CARB levels and MetS. Interaction terms were tested for each variable to investigate effect modifiers, but none were significant. Median P_CARB values for each quartile were used as continuous variables to calculate trend *p*-values. Three analytical models were constructed: Model 1: unadjusted; Model 2: adjusted for age, sex, and BMI; and Model 3: further adjusted for household income; physical activity; smoking status; alcohol consumption; total energy intake; carbohydrate quality score; and baseline levels of fasting blood glucose, total cholesterol, and systolic and diastolic blood pressure.

Restricted cubic spline regression was employed to explore potential non-linear relationships between the P_CARB levels and the risk of MetS and its components. This method allows for flexible modeling of non-linear relationships, enhancing our ability to capture complex patterns in the data. Three knots were placed at the 5th, 50th, and 95th percentiles of the P_CARB distribution to ensure a good fit across the data range while avoiding overfitting. All statistical tests were conducted at a significance level of α = 0.05, using SAS version 9.4 (SAS Institute Inc., Cary, NC, USA).

## 3. Results

The mean follow-up period of this study was 9.54 years. Among the 7902 participants, there were a total of 2785 cases of MetS.

### 3.1. General Characteristics of the Participants According to P_CARB

Table 1 shows the baseline characteristics of the participants categorized by quartiles of the P_CARB. The mean values of P_CARB for the lowest quartile (Q1) and the highest quartile (Q4) were 63.57% and 78.74%, respectively. Participants in Q4 had a higher proportion of women, older individuals, and those with lower household incomes. This group also had a greater number of non-smokers, non-drinkers, and individuals with high physical activity levels, and exhibited lower total energy intake compared to those in Q1 (*p* < 0.001). However, the P_CARB was not significantly correlated with BMI (*p* = 0.9).

### 3.2. Association between P_CARB and MetS

Table 2 presents the HRs for the risk of developing various components of MetS, based on quartiles of the P_CARB. Higher P_CARB was associated with a greater risk of hypertriglyceridemia (*p* for trend = 0.002), hypo-HDL cholesterolemia (*p* for trend < 0.001), dyslipidemia (*p* for trend = 0.002), high blood pressure (*p* for trend = 0.01), and MetS (*p* for trend = 0.02). In addition, individuals in the highest quartile (Q4) of carbohydrate intake showed significantly increased risks for hypertriglyceridemia (HR 1.25, 95% CI: 1.09–1.44), hypo-HDL cholesterolemia (HR 1.28, 95% CI: 1.15–1.43), dyslipidemia (HR 1.14, 95% CI: 1.04–1.24), high blood pressure (HR 1.14, 95% CI: 1.03–1.25), and MetS (HR 1.17, 95% CI: 1.02–1.33). However, high fasting glucose did not show a significant trend in the fully adjusted model, nor in the comparison between quartiles (HR 0.92, 95% CI: 0.81–1.03, *p* for trend = 0.1).

### 3.3. Dose–Response Relationship between Proportion of Total Energy from Carbohydrate Intake and the Risk of MetS

**Figure 2:**
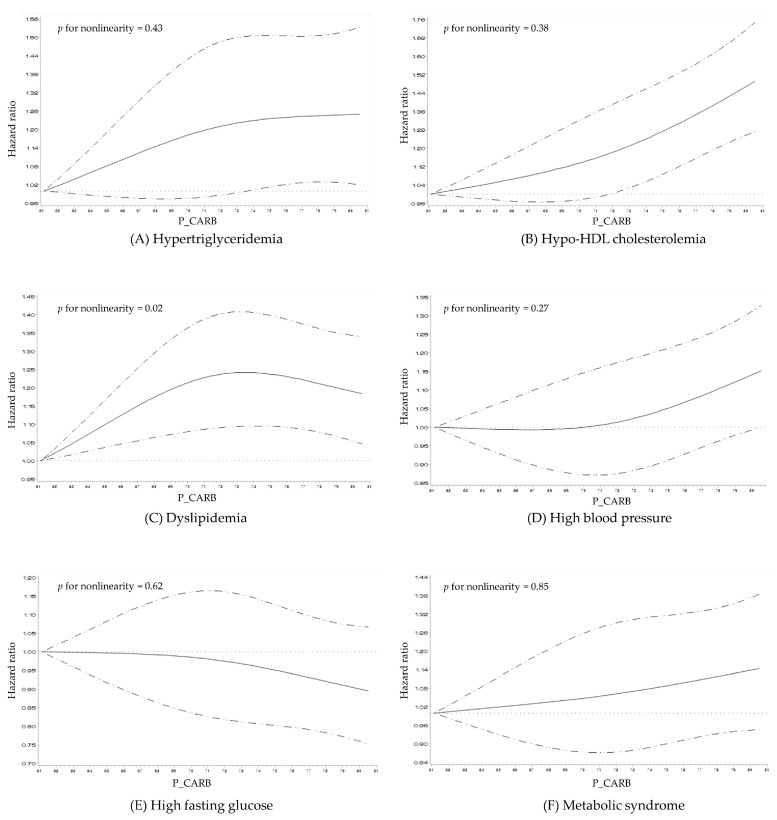
Multivariable adjusted hazard ratios (95% confidence intervals) for the dose–response relationship between the proportion of total energy from carbohydrates and metabolic disease risk in middle-aged Korean adults. The model is adjusted for age; sex; household income level; smoking status; alcohol consumption; physical activity level; body mass index; total energy intake; carbohydrate quality sore; and baseline levels of fasting blood glucose, total cholesterol, and systolic and diastolic blood pressure. P_CARB, proportion of total energy from carbohydrate. (**A**) Hypertriglyceridemia, (**B**) Hypo-HDL cholesterolemia, (**C**) Dyslipidemia, (**D**) High blood pressure, (**E**) High fasting glucose, (**F**) Metabolic syndrome.

Figure 2 illustrates the dose–response relationship between the P_CARB and the risk of MetS. After adjusting for all covariates included in Model 3 (refer to Table 2), the spline curve revealed a linear increase in the risk of hypertriglyceridemia, hypo-HDL cholester-olemia, high blood pressure, and MetS as the P_CARB increased (*p* for nonlinearity: hypertriglyceridemia = 0.43, hypo-HDL cholesterolemia = 0.38, high blood pressure = 0.27, MetS = 0.85). In contrast, dyslipidemia exhibited an inverse U-shaped relationship with the P_CARB, indicating a non-linear risk pattern. Additionally, high fasting glucose demonstrated a linear decrease in risk with increasing P_CARB (*p* for nonlinearity = 0.62); however, the association shape appears relatively flat.

## 4. Discussion

This study analyzed the longitudinal association between the P_CARB and MetS in the KoGES, a large-scale epidemiological study of middle-aged Korean adults. The results indicated that as the P_CARB increased, the risk of hypertriglyceridemia, hypo-HDL cholesterolemia, high blood pressure, and MetS also increased, demonstrating a positive dose–response relationship. 

According to a cross-sectional study analyzing the association between carbohydrate intake and metabolic risk factors based on the National Health and Nutrition Examination Survey data from two different countries, Korea and the US, a significant positive association was found only in the Korean population [9]. The mean of P_CARB was 81.5% in Korea and 65.8% in the US, with 57% of individuals in Korea exceeding the acceptable macronutrient distribution range of 65%, compared to only 8% in the US, indicating a marked difference in the level of carbohydrate intake between the two countries [9]. From 2015 to 2016, American adults obtained approximately 50% of their daily energy from carbohydrates, while Koreans obtained approximately 66% during the same period. Carbohydrate intake in Koreans is substantially higher than in other East Asian countries, such as China (55%) and Japan (60%) [21,22,23,24]. In Western countries like the US, carbohydrates are not a staple food and differ from the types of carbohydrates that Koreans mainly consume.

A meta-analysis using 14 cross-sectional studies and 4 cohort studies showed that the prevalence of MetS was 1.25 times higher in the group with the highest carbohydrate intake compared to the lowest intake group. Dose–response analysis also showed a linear association between carbohydrate intake and MetS [25]. Furthermore, a meta-analysis including 432,179 participants from 8 studies demonstrated a significant association between high carbohydrate consumption and mortality related to cardiac metabolic diseases, including hypertension [26]. Consumption of carbohydrates, especially simple sugars, can temporarily elevate blood pressure and increase insulin secretion, which has an antidiuretic effect, reducing sodium excretion and further elevating blood pressure [27].

The adverse effects of excessive carbohydrate intake on cardiometabolic health risks can be explained by several mechanisms. Consuming excessive carbohydrates promotes insulin resistance, leading to increased fat accumulation on blood vessel walls; limits salt excretion in the kidneys; and enhances vascular smooth muscle contraction, thereby raising the risk of hypertension [28,29]. Additionally, the body’s cholesterol pool is maintained through synthesizing lipids in various body tissues or obtaining lipids from foods, which are absorbed through the epithelial cells of the small intestine [30]. The liver converts excess carbohydrates into fat via de novo lipogenesis and releases them into the bloodstream [31,32,33]. Elevated triglyceride levels caused by metabolic problems and poor lifestyles are independently associated with coronary artery disease and are factors that increase the risk of MetS [34]. As such, the results of this study show that serum triglyceride and HDL cholesterol, which directly affect blood health, are directly related to carbohydrate intake.

Carbohydrates containing a large number of dietary fiber tend to have a reverse association with MetS and the risk of heart disease. Several studies have found that increased consumption of carbohydrate-rich foods, including cereals, whole grains, fruits, vegetables, and other fiber-rich foods, is associated with reductions in body weight, serum cholesterol levels, and systolic blood pressure [35,36]. Therefore, we adjusted for carbohydrate quality to minimize confounding from high-quality carbohydrates with high dietary fiber and those with low dietary fiber. However, despite this adjustment, we still found an association between high P_CARB and metabolic disease in the Korean population. This may be because the proportion of individuals consuming high-fiber carbohydrates is not large enough to offset the effects of low-fiber, low-quality carbohydrates, such as white rice, which are commonly consumed as staple foods in Korea. These low-fiber carbohydrates are more strongly associated with metabolic diseases [24,37]. Further clinical studies on the effects of different types of carbohydrates on metabolic diseases, especially in populations with high carbohydrate intake, are needed.

This study has several strengths. One significant strength is its longitudinal design, which analyzes the relationship between a high-carbohydrate diet and the risk of metabolic risk factors in a large cohort of middle-aged Korean adults. Although dietary intake was not repeatedly measured, the longitudinal nature of the study helps to observe the development of metabolic risk factors over time. The study also comprehensively adjusts for a wide range of potential confounding factors, including age, sex, household income level, smoking status, alcohol consumption, physical activity level, BMI, total energy intake, and carbohydrate quality score. This comprehensive adjustment helps to isolate the effect of carbohydrate intake on metabolic outcomes. Lastly, the SQFFQ used in the study is a validated tool designed to reflect the dietary habits of middle-aged Koreans, and averaging data from two dietary assessments further minimizes potential errors related to recent dietary intake.

However, there are limitations to this study. Despite adjusting for various potential confounders, there may still be residual confounding factors that have not been measured or scientifically defined, which could influence the observed associations. Additionally, the study focuses on middle-aged Korean adults, which may limit the generalizability of the results to other age groups or populations with different dietary patterns and lifestyle factors. Although the study adjusted for carbohydrate quality, the analysis might still be influenced by the predominant consumption of low-quality carbohydrates, such as white rice, which are common in the Korean diet, affecting the interpretation of the association between high carbohydrate intake and metabolic risks. In addition, the carbohydrate-to-fiber ratio does not account for the differences between various types of carbohydrates, such as fructose and starch. Fructose and starch have distinct metabolic effects and health implications. Furthermore, dietary habits and lifestyle factors may change over time, which can influence the long-term associations observed in the study, and the study may not fully account for these temporal changes.

## 5. Conclusions

Our findings suggest that a high-carbohydrate diet may adversely affect metabolic risk factors, potentially increasing the risk for CVD and other chronic health conditions. These results underscore the importance of maintaining a balanced diet that aligns with the acceptable macronutrient distribution range for carbohydrates while adopting healthy lifestyle habits. Our research supports the notion of reducing the P_CARB and increasing the intake of proteins and lipids for middle-aged Korean adults who currently consume a high-carbohydrate diet. Furthermore, future intervention studies should explore how changes in carbohydrate intake within the Korean population influence the association between the P_CARB and various health outcomes, considering broader age groups to promote comprehensive health benefits.

## Figures and Tables

**Table 1:** Baseline characteristics of participants categorized by quartiles of carbohydrate energy proportion intake.

	Quartile of P_CARB				*p* Value ^1^
	Q1	Q2	Q3	Q4	
No. of participants	1975	1976	1976	1975	
P_CARB (%)	63.57 ± 0.05	69.95 ± 0.05	73.79 ± 0.05	78.74 ± 0.05	
Sex					<0.001
Men	1081 (54.73)	1031 (52.18)	886 (44.84)	658 (33.32)	
Women	894 (45.27)	945 (47.82)	1090 (55.16)	1317 (66.68)	
Age					<0.001
40–49	1300 (65.82)	1118 (56.58)	951 (48.13)	616 (31.19)	
50–59	420 (21.27)	497 (25.15)	519 (26.27)	545 (27.59)	
≥60	255 (12.91)	361 (18.27)	506 (25.60)	814 (41.22)	
Household income (KRW) ^2^					<0.001
Low or mid-low	657 (33.62)	770 (39.43)	1008 (51.91)	1404 (72.82)	
Mid-high or high	1297 (66.38)	1183 (60.57)	934 (48.09)	524 (27.18)	
Smoking status					<0.001
Smokers	631 (32.41)	543 (27.69)	444 (22.65)	369 (19.02)	
Non-smokers	1316 (67.59)	1418 (72.31)	1516 (77.35)	1571 (80.98)	
Alcohol consumption					<0.001
Drinkers	1163 (59.16)	1050 (53.46)	918 (46.72)	631 (32.31)	
Non-drinkers	803 (40.84)	914 (46.54)	1047 (53.28)	1322 (67.69)	
Physical activity levels ^3^					<0.001
Low	676 (34.65)	701 (35.69)	649 (33.10)	572 (29.47)	
Moderate	724 (37.11)	730 (37.17)	664 (33.86)	499 (25.71)	
High	551 (28.24)	533 (27.14)	648 (33.04)	870 (44.82)	
Total energy intake (kcal/day)	2129.95 ± 11.23	1919.94 ± 11.23	1787.22 ± 11.23	1640.29 ± 11.23	<0.001
Body mass index (kg/m^2^)	24.08 ± 0.07	24.08 ± 0.07	24.03 ± 0.07	24.04 ± 0.07	0.9
Serum total cholesterol (mg/dL)	192.44 ± 0.79	191.30 ± 0.79	187.26 ± 0.79	187.42 ± 0.79	<0.001
Serum HDL cholesterol (mg/dL)	47.05 ± 0.22	46.34 ± 0.22	46.03 ± 0.22	45.72 ± 0.22	<0.001
Serum triglycerides (mg/dL)	130.57 ± 1.87	129.05 ± 1.87	131.37 ± 1.87	132.95 ± 1.87	0.5
LDL cholesterol (mg/dL) ^4^	119.32 ± 0.73	119.15 ± 0.73	114.95 ± 0.73	115.11 ± 0.73	<0.001

Q, quartile; P_CARB: proportion of total energy from carbohydrates; KRW, Korean Republic Won; HDL, high density lipoprotein; LDL, low density lipoprotein. Values are n (%) or mean ± standard error. ^1^ *p* value for the differences in variables according to the quartile of the P_CARB. ^2^ Household income was divided into <1.5 million KRW (low or mid-low) and ≥1.5 million KRW (mid-high or high). ^3^ Physical activity level was calculated as METs-hours/week and categorized into tertiles. ^4^ LDL cholesterol levels were estimated using the Friedewald formula.

**Table 2:** Hazard ratios (95% confidence intervals) for metabolic syndrome, stratified by quartiles of carbohydrate energy proportion intake.

	Quartile of P_CARB				*p* for Trend
	Q1	Q2	Q3	Q4	
Hypertriglyceridemia					
No. of cases (%)	584 (29.57)	620 (31.38)	595 (30.11)	611 (30.94)	
Model 1	1	1.04 (0.93–1.17)	0.99 (0.88–1.11)	1.03 (0.92–1.16)	0.8
Model 2	1	1.05 (0.94–1.18)	1.04 (0.93–1.17)	1.15 (1.02–1.29)	0.04
Model 3	1	1.11 (0.98–1.25)	1.14 (1.00–1.29)	1.25 (1.09–1.44)	0.002
Hypo-HDL cholesterolemia					
No. of cases (%)	895 (45.32)	930 (47.06)	1021 (51.67)	1046 (52.96)	
Model 1	1	1.06 (0.97–1.17)	1.20 (1.09–1.31)	1.28 (1.17–1.40)	<0.001
Model 2	1	1.07 (0.98–1.18)	1.24 (1.13–1.35)	1.36 (1.24–1.50)	<0.001
Model 3	1	1.06 (0.97–1.17)	1.19 (1.07–1.31)	1.28 (1.15–1.43)	<0.001
Dyslipidemia					
No. of cases (%)	1545 (78.23)	1589 (80.41)	1610 (81.48)	1606 (81.32)	
Model 1	1	1.06 (0.99–1.14)	1.08 (1.01–1.16)	1.10 (1.03–1.18)	0.006
Model 2	1	1.05 (0.98–1.13)	1.06 (0.99–1.14)	1.07 (0.99–1.15)	0.08
Model 3	1	1.08 (1.01–1.17)	1.13 (1.05–1.22)	1.14 (1.04–1.24)	0.002
High blood pressure					
No. of cases (%)	1095 (55.44)	1115 (56.43)	1210 (61.23)	1347 (68.20)	
Model 1	1	1.01 (0.93–1.10)	1.16 (1.07–1.26)	1.45 (1.34–1.57)	<0.001
Model 2	1	0.95 (0.87–1.03)	1.03 (0.95–1.12)	1.13 (1.04–1.23)	0.001
Model 3	1	0.96 (0.88–1.05)	1.01 (0.92–1.10)	1.14 (1.03–1.25)	0.01
High fasting glucose					
No. of cases (%)	818 (41.42)	827 (41.85)	786 (39.78)	810 (41.01)	
Model 1	1	0.99 (0.90–1.09)	0.90 (0.82–1.00)	0.94 (0.85–1.04)	0.1
Model 2	1	0.99 (0.90–1.09)	0.90 (0.82–1.00)	0.94 (0.85–1.04)	0.1
Model 3	1	0.96 (0.87–1.06)	0.92 (0.83–1.03)	0.92 (0.81–1.03)	0.1
Metabolic Syndrome					
No. of cases (%)	644 (32.61)	664 (33.60)	706 (35.73)	771 (39.04)	
Model 1	1	1.01 (0.91–1.12)	1.10 (0.99–1.23)	1.29 (1.16–1.43)	<0.001
Model 2	1	0.98 (0.88–1.09)	1.05 (0.94–1.17)	1.16 (1.04–1.30)	0.005
Model 3	1	1.01 (0.90–1.13)	1.08 (0.96–1.22)	1.17 (1.02–1.33)	0.02

Q, quartile; HDL, high density lipoprotein. Model 1: unadjusted. Model 2: adjusted for age, sex, and body mass index. Model 3: Model 2 plus additional adjustments for the household income level; smoking status; alcohol consumption; physical activity level; total energy intake; carbohydrate quality sore; and baseline levels of fasting blood glucose, total cholesterol, and systolic and diastolic blood pressure.

## Data Availability

The KoGES data used in this study are available upon request and subject to approval. Interested researchers may apply for access via the National Institute of Health Korea website (https://www.nih.go.kr), where applications will be reviewed and approved accordingly.
